# Subconjunctival ocular filariasis -Case report-

**DOI:** 10.22336/rjo.2017.14

**Published:** 2017

**Authors:** Sorin Simion Macarie, Cristina Dobre, Marilena-Cristina Suciu, Angela-Monica Ionica, Mihai-Sorin Cernea, Paul Tarcău, Flaviu Bodea

**Affiliations:** * Department of Ophthalmology, “Iuliu Hatieganu” University of Medicine and Pharmacy, Cluj-Napoca, Romania; ** County Emergency Hospital, Cluj-Napoca, Romania; *** Faculty of Veterinary Medicine, University of Agricultural Sciences and Veterinary Medicine, Cluj-Napoca, Romania

**Keywords:** Dirofilaria repens, ocular dirofilariasis, ocular parasite

## Abstract

We are presenting the case of a patient who was clinically diagnosed with subconjunctival ocular dirofilariasis, confirmed by the parasitological examination. The treatment consisted in the surgical extraction of the parasite, a local treatment with antibiotics and steroidal anti-inflammatory mydriatic and general treatment with antihelminthic, antibiotic, analgesic, and anti-inflammatory drugs. The intraoperative and postoperative evolution of the case was favorable.

## Introduction

Dirofilaria repens (Spirurida, Onchocercidae) is a nematode that parasitizes mainly dogs (Canis lupus familiaris) and other mammals, but may also infect humans, being considered a zoonotic agent [**1**]. The parasite’s most frequent localization in humans is in subcutaneous and ocular tissue (75.8%), especially in the ocular area, which is accessible to mosquitoes that act as vectors [**2**].

Adult parasites are found in subcutaneous tissues while the larvae (known as microfilariae) are found in the blood of the infested animals. They are ingested by mosquitoes of genera Aedes, Anopheles, or Culex during the blood meal. The larvae grow and become infective inside the mosquito’s body. Infective L3 larvae may be transferred to humans through inoculation when the mosquitoes feed [**3**].

## Case report

A 54-year-old female patient, living in a rural area in Salaj county, Romania, having contact with dogs, cats, pigs, rabbits in the household, presented to the emergency room of Cluj Ophthalmology Clinic, complaining of a sudden ocular pain that persisted from the previous day, with burning, itching and epiphora in the left eye (LE).

Family history and personal history were not relevant to the condition for which the patient presented to our clinic.

Functional ocular examination revealed a visual acuity of 20/ 20 in both eyes, normal intraocular pressure (14 mmHg in the right eye (RE) and 17 mmHg in the LE).

A round formation containing a mobile larva in the subconjunctival temporal region of bulbar conjunctiva was observed at the slit lamp examination of the LE, overlying a conjunctival congestion and underlying the episcleral tissue.

Examination of the fundus of the eye revealed a well-defined vital papilla, a macula with reduced foveolar reflex, and normal blood vessels without the presence of other larval forms at the back of the eyeball.

Based on clinical examination, the LE diagnose was subconjunctival ocular parasitosis. General clinical examination did not reveal the presence of subcutaneous nodules, which might be also present in Dirofilaria repens infestation. Heart ultrasound, abdominal ultrasound, and chest X-ray showed normal relations.

Laboratory examinations: blood picture with unimportant changes, normal liver enzymes, creatinine, glucose, cholesterol, triglycerides and coagulation, increased fibrinogen (520.3 mg/ dl, VN: 200-400 mg/ dl), CIC U x 10 ^ 95 3 normal C3, C4 slightly increased, IgA and IgG normal, IgM slightly decreased.

**Fig. 1:**
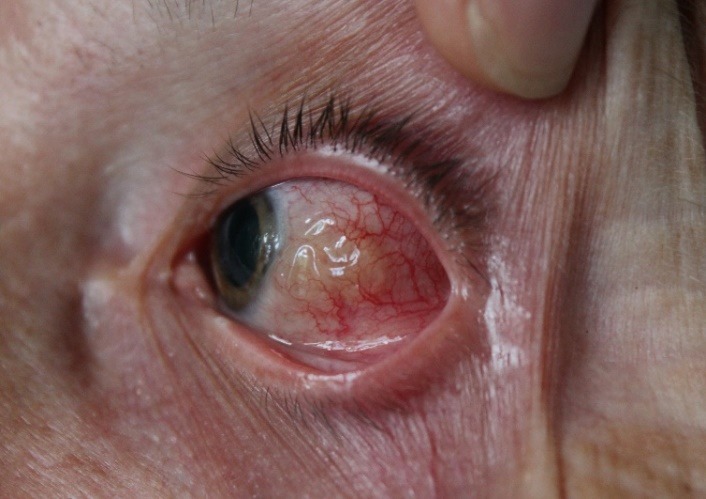
The appearance of the subconjunctival parasite

**Fig. 2:**
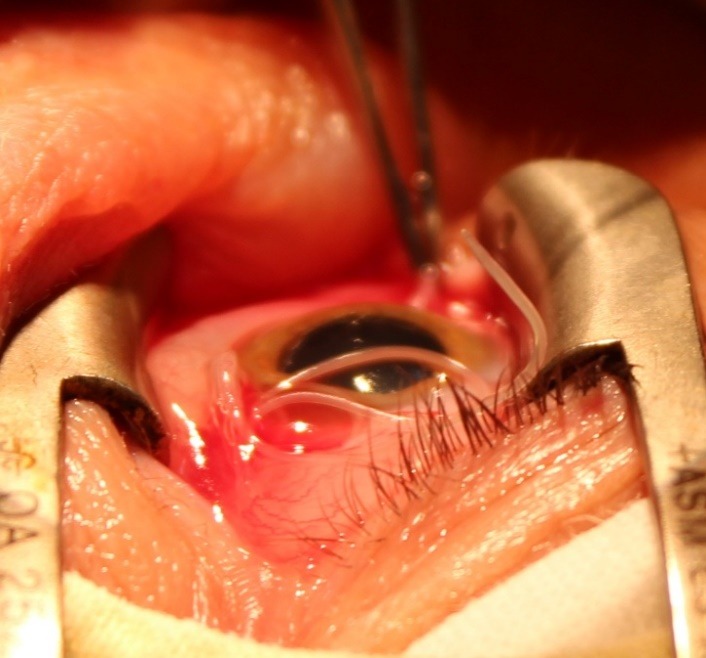
The surgical extraction of the parasite

**Fig. 3:**
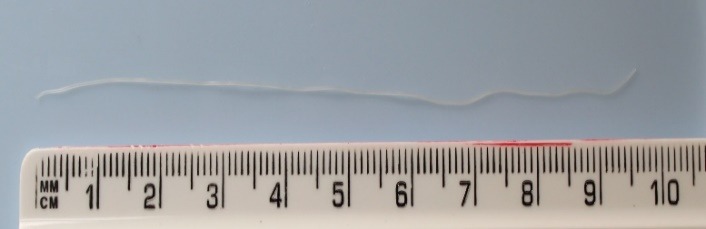
Measurement of the parasite

We decided to surgically extract the parasite. The surgery resulted in the extraction of a white, translucent parasite with a length of about 10 cm and a diameter of about 0.5 mm (**Fig. 1**-**3**). Both the surgical and the postsurgical evolution were favorable.

The parasitological examination revealed that the parasite was an immature male of Dirofilaria repens (L5). Nematode identification was based on morphological characters described in the literature: rounded ends, the presence of longitudinal cuticular ridges, the shape and arrangement of caudal papillae [**3**,**4**] (**Fig. 6**); sex was determined by emphasizing the male genitalia (spikes) which were not yet fully developed (**Fig. 5**,**6**).

During hospitalization, the patient received treatment with local antibiotic, anti-inflammatory steroid, mydriatics and general treatment with antibiotics, pain relievers, and anti-inflammatories. After the identification of the parasitic species, the patient received treatment with 400 mg albendazole two times daily, for three days.

No short term or long-term complications were noticed in the evolution of this patient.

**Fig. 4:**
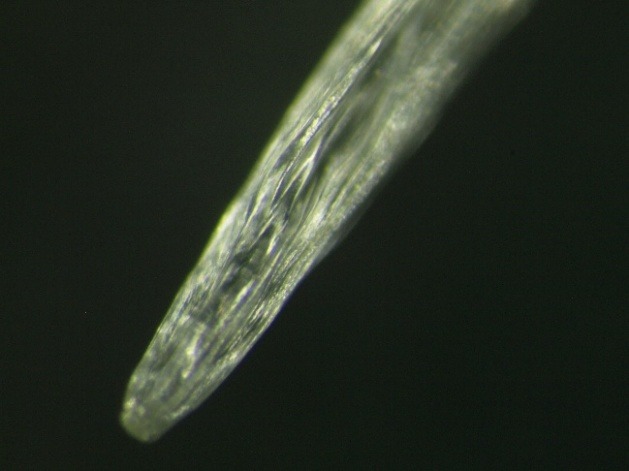
The appearance of the anterior extremity of the Dirofilaria repens (x20)

**Fig. 5:**
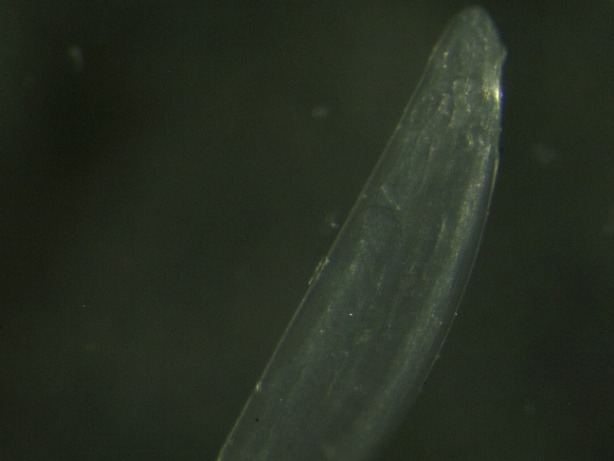
The appearance of Dirofilaria repens posterior end with spikes visible (x20)

**Fig. 6:**
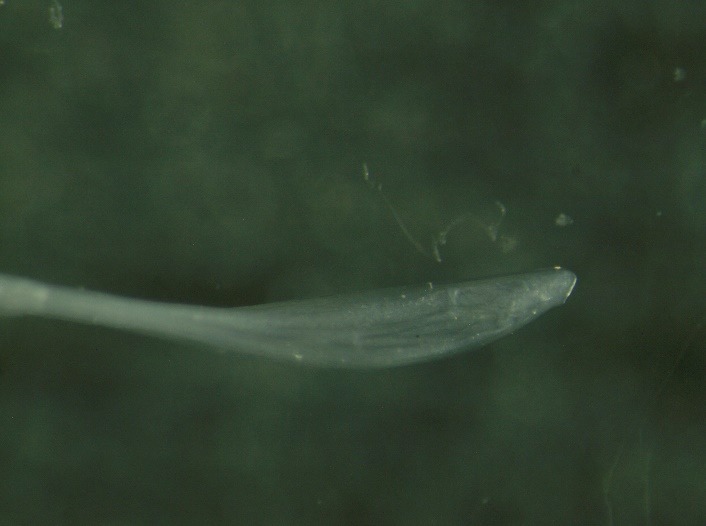
Dirofilaria repens posterior end with spikes visible (x20)

**Fig. 7:**
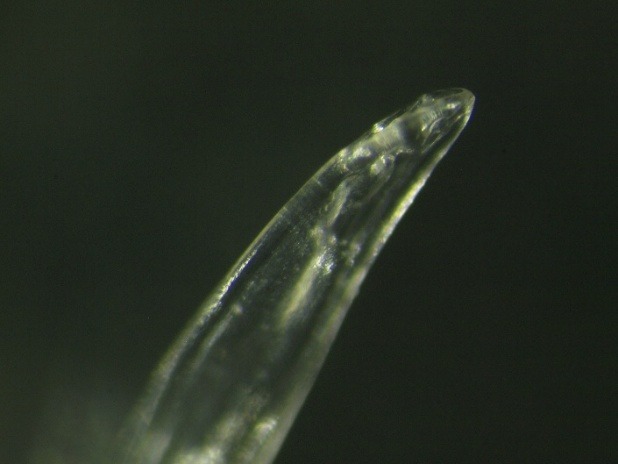
Dirofilaria repens posterior end, caudal papillae (x20)

## Conclusions

The geographical distribution of Dirofilaria repens has changed considerably in recent decades. Before 2001, the area mainly consisted of scattered areas of Italy, Greece, Spain, and southern France [**5**]. In correlation with a variety of factors, mainly climate change, this area has expanded into many countries in Central, Northeast, and East Europe, including Romania [**6**,**7**].

Similar to canine heartworm (D. immitis), most human cases in Europe were recorded in Italy and France [**2**]. There is a positive correlation between the prevalence of infestation in canine species and the risk of human infection [**8**]. Sporadic cases have been reported in Belgium, Bulgaria, Greece, Romania, Russia, Serbia, Slovakia, Slovenia, Spain, Ukraine, and Hungary [**2**,**9**].

After 2005, 16 other human cases have been reported in Romania [**10**-**14**], most of which involved patients living in the SE of the country, which correlates with local climatic conditions and the high infestation rates that were found in dogs from that area [**15**].

In the past, it was thought that man is an accidental and terminal host for this parasite and that full development cannot be achieved in the human body. More recent studies based on the discovery of microfilariae in subcutaneous nodules in humans, suggested, however, that man would be a favorable host for reaching sexual maturity [**16**]. In the clinical presented case, the immature parasite was located under the conjunctiva of the eye, without the presence of any subcutaneous nodules being revealed.

Previous studies have revealed high levels of specific IgG in the serum of patients infected with Dirofilaria repens [**17**]. In the subcutaneous forms, they are described by the presence of 26-40 kDa polypeptide fragments belonging to adult parasite antigenic complex in serum and changes in blood picture, with increased eosinophilia [**17**,**18**]. The presented case showed insignificant changes in the blood picture, the eosinophilia being within normal limits. Given that a single male parasite, that did not reach sexual maturity, was revealed under the conjunctiva of the eye, it was not considered necessary to carry out a determination of specific IgG or measurements of the antigenic fragments from serum. There is no data in literature to correlate the changes in fibrinogen, C4, IgM with the nematode’s presence.
